# Disabling pansclerotic morphea of childhood – unusual case and management challenges

**Published:** 2008-08-15

**Authors:** Forsea Ana-Maria, Cretu Aura-Nicoleta, Ionescu Ruxandra, Giurcaneanu Calin

**Affiliations:** * Carol Davila University of Medicine and Pharmacy– Department of Oncologic Dermatology and Allergology, Elias University Emergency Hospital, Bucharest; ** Carol Davila University of Medicine and Pharmacy- Department of Internal Medicine and Rheumatology, Sf. Maria Hospital , Bucharest

**Keywords:** disabling pansclerotic morphea of childhood, scleroderma, methylprednisolone pulse-therapy, methotrexate, UVA

## Abstract

Morphea, also known as *localized scleroderma* is a chronic disease of unknown etiology, characterized by fibrous deposition and obliteration of vessels in the skin. This disease has a wide clinical spectrum, ranging from mild hyperpigmented plaques to severe, invalidating generalized and pansclerotic forms. Disabling pansclerotic morphea of childhood is a rare and debilitating variant of localized scleroderma, characterized by a rapid progression of deep cutaneous fibrosis that involves the dermis and the subcutaneous adipose tissue but also fascia, muscles, and bone. Contractures and musculoskeletal atrophy develop and the disease has an invalidating and even fatal course.

We present an unusual case of severe morphea in a 19-year-old girl, with a polymorphous clinical picture consisting of plaques, linear and pansclerotic, circumferential lesions, with symmetric, invalidating involvement of all limbs and explosive evolution with centripetal progression. This case emphasizes the unpredictable character of morphea evolution, the possible severe prognosis and the therapeutic challenges raised by the generalized, disabling forms of this disease.

## Introduction

Morphea, also referred to as *localized scleroderma or circumscribed scleroderma* represents a localized cutaneous sclerosis, characterized by the obliteration of the cutaneous small vessels and capillaries and fibrous and degenerative lesions of the skin. It is a chronic disease which manifests clinically by initially inflammatory, violaceous plaques, that later become indurate and atrophic. The fibrotic process may progress to different depths, involving the dermis, the subcutaneous fat, and sometimes even the underlying soft tissues and bone.

Localized scleroderma has a wide clinical spectrum, ranging from superficial, circumscribed sclerotic plaques to severe, generalized and pansclerotic forms. The numerous clinical variants include: plaque-type morphea - including superficial, guttate and nodular variants; generalized morphea; linear scleroderma - including “en coup de sabre” lesions and Parry-Romberg syndrome (progressive hemi facial atrophy); and deep morphea - including morphea profunda, eosinophilic fasciitis and disabling pansclerotic morphea of childhood (DPMC) [**1**, **2**]. 

No matter the clinical form of morphea, the etiology is still unknown. The pathogenic mechanisms of this disease include: i. injury of the endothelial cells; ii. abnormal activation of the immune response; iii. hyperactivity of fibroblasts with excessive collagen synthesis [**3**]. Genetic background with predisposition to autoimmune disorders, immunologic alterations and environmental factors such as trauma, toxins exposure or infections (especially infection with *Borrelia burgdorferi*) are considered to play a role in the development of morphea. Localized scleroderma lacks visceral involvement and must be distinguished from cutaneous sclerosis occurring in systemic diseases such as progressive systemic sclerosis or graft-versus-host disease.

Disabling pansclerotic morphea of childhood is a rare and severe type of deep morphea, which usually begins under the age of 14 and is characterized by rapidly evolving sclerosis involving all the layers of the skin, but also the fascia, muscles, and bone (pansclerosis) [**4**]. It leads to muscle and skeletal atrophies, contractions and articular ankylosis [**5**]. Beyond the handicap produced by ankylosis, the prognosis of this disease on longer term is shadowed by the late complications such as trophic cutaneous ulcers, nerve compressions and cutaneous carcinoma. Secondary skin neoplasia, septicemia and restrictive pulmonary disease carry a vital risk [**6**, **7**].

 An efficient therapy of morphea is not defined yet. The disease has a chronic unpredictable evolution ranging from rare spontaneous resolution to rapidly progressing, generalized, life-threatening forms. Attempts to stop progression and revert skin fibrosis have been made with only moderate success using a wide range of medications including corticosteroids (topical, intralesional and systemic); vitamin D analogs; antibiotics; antimalarials; D-penicillamine; colchicine; methotrexate; retinoids and many others [**8**, **9**, **10**].

## Case presentation

**Fig.1:**
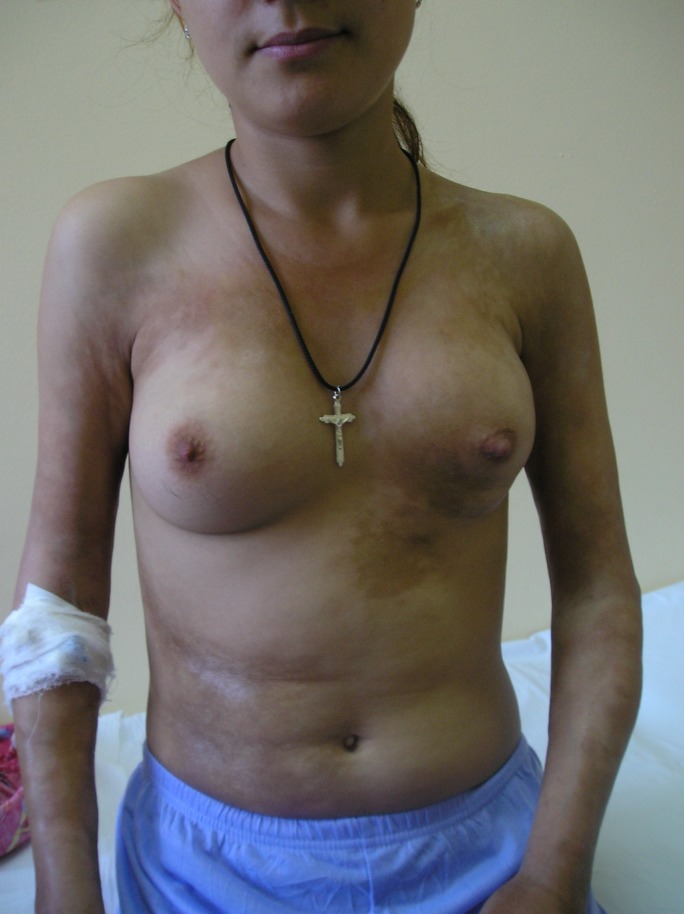
disseminated hyperpigmented sclero-atrophic plaques on the chest and flanks, moderate atrophy of left breast caused by fibrotic process and discrete limitation of the extension of elbows (maximal 150 degrees)

**Fig.2:**
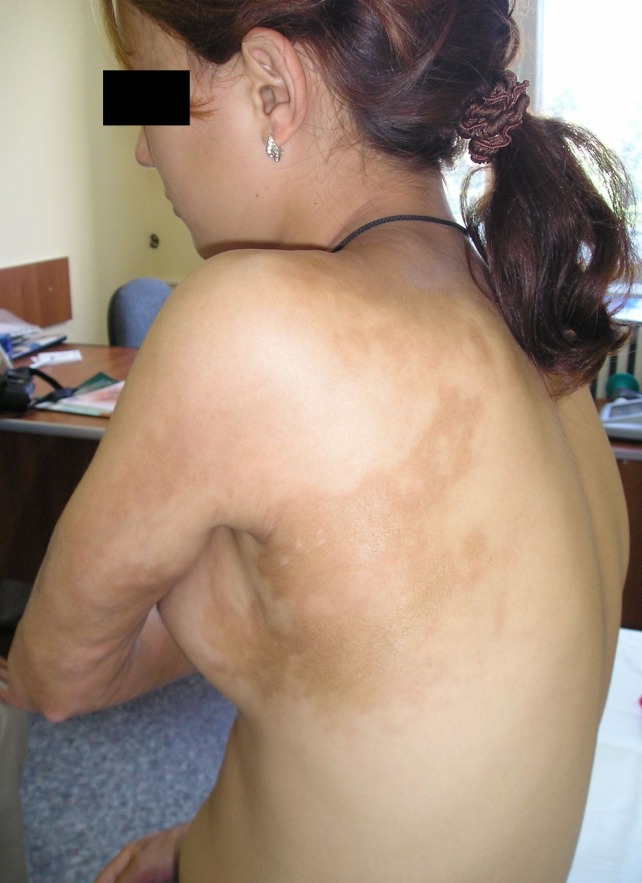
linear sclero-atrophic lesions on posterior left hemitorax, following Blashko’s lines and on posterior side of left arm

**Fig.3:**
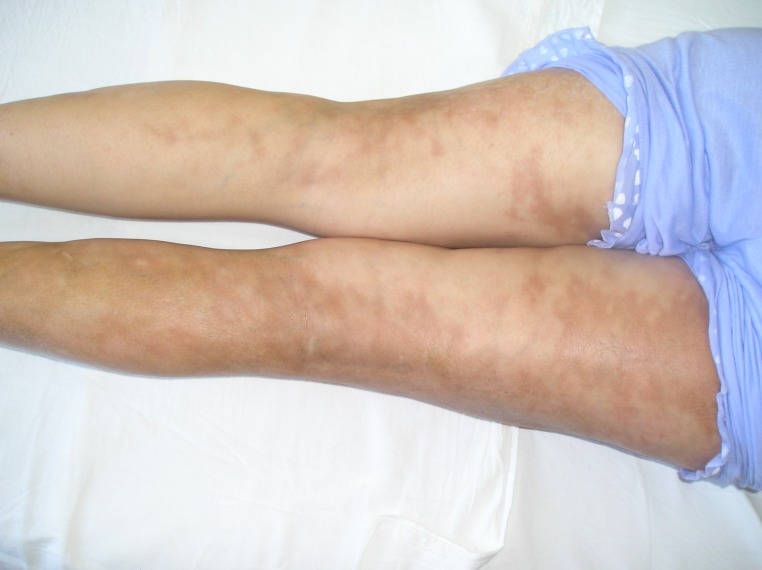
delineation of linear lesions on both inferior limbs

We present the case of a 19-year-old girl with an atypical presentation of disabling pansclerotic morphea. The disease onset occurred at the age of 12 with a unique sclero-atrophic plaque in the left hypochondrium. Over the following 5 years the condition progressed slowly, with new sclerotic plaques developing on the chest and flanks, followed by the appearance of linear scleroderma lesions involving the limbs and left hemithorax. The diagnosis of morphea was first established at the age of 15 and attempts of therapy with local corticosteroids had no result. At the age of 17 (January 2006), the patient presented to our clinic with a polymorphous clinical picture consisting in disseminated polymorphous sclero-atrophic plaques and linear lesions on arms and legs and on the chest and flanks, following Blashko’s lines, (Figures 1-3) with discrete limitation of the extension of the elbows (maximal 150 degrees). General clinical examination did not reveal any signs of visceral involvement. Sclerodactylia and Raynaud phenomenon were absent. Laboratory work-up showed moderate hypochromatic anemia (Hb=10,9g/dL), and elevated erythrocyte sedimentation rate (ESR=40mm/1h) with no further abnormality. Immunologic profile was normal, antinuclear antibodies (ANA), anti-Scl70 and anti-centromere antibodies were absent. *Borrelia burgdorferi* serology was negative, and there was no history of toxic exposure. Thus the diagnosis of morphea was confirmed. 

Under systemic treatment using low doses of prednisone (20mg/day, slowly tapered), colchicine, penicillin (100000UI/day), antioxidants, and pentoxyfillin, associated with physiotherapy, the disease was stabilized, with no occurrence of new lesions, discrete softening of the existing ones and discrete increase of the mobility of the involved joints. In the same time, over 3 months, ESR decreased to normal range.

After 4 months of the mentioned treatment, the patient developed an infection of the upper respiratory airways, managed in ambulatory setting. Reportedly, the acute episode was followed by symptoms of migratory arthritis, with tumefaction, erythema and pain in the left knee, shifting to the left ankle and associated with a slight reactivation of scleroderma lesions.

Subsequently, the patient self-decided the discontinuation of the monitoring in our clinic, as well as all treatment for morphea. She took up a naturist-prescribed detoxification diet based only on fruits and vegetables.

**Fig.4:**
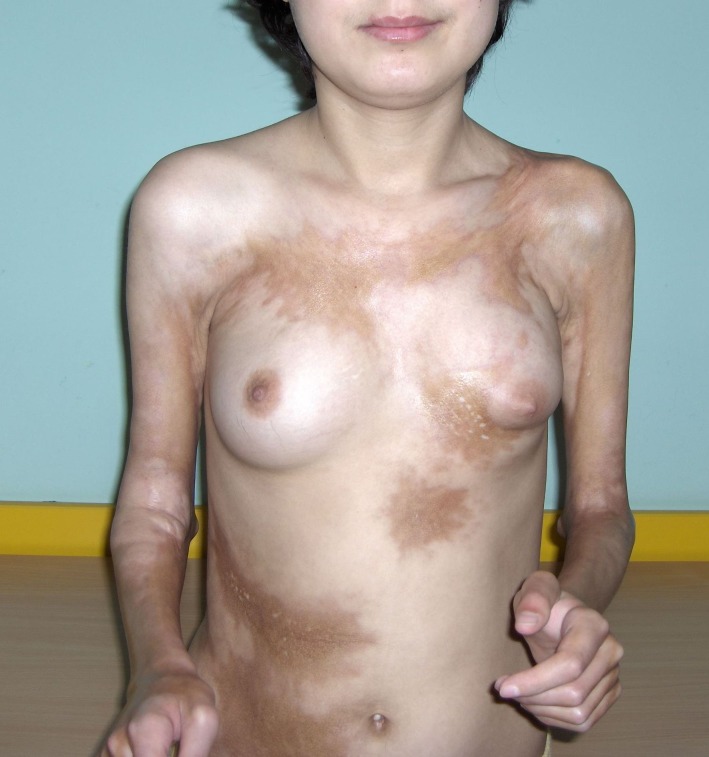
extended hyperpigmented sclero-atrophic plaques on flanks and chest, with indurations and severe atrophy of the left breast and sclerosis involving all the surface of forearms and elbows up to distal third of the arms; fixation of elbows at 90 degrees flexion; fibrous bands extended to the left shoulder

**Fig.5:**
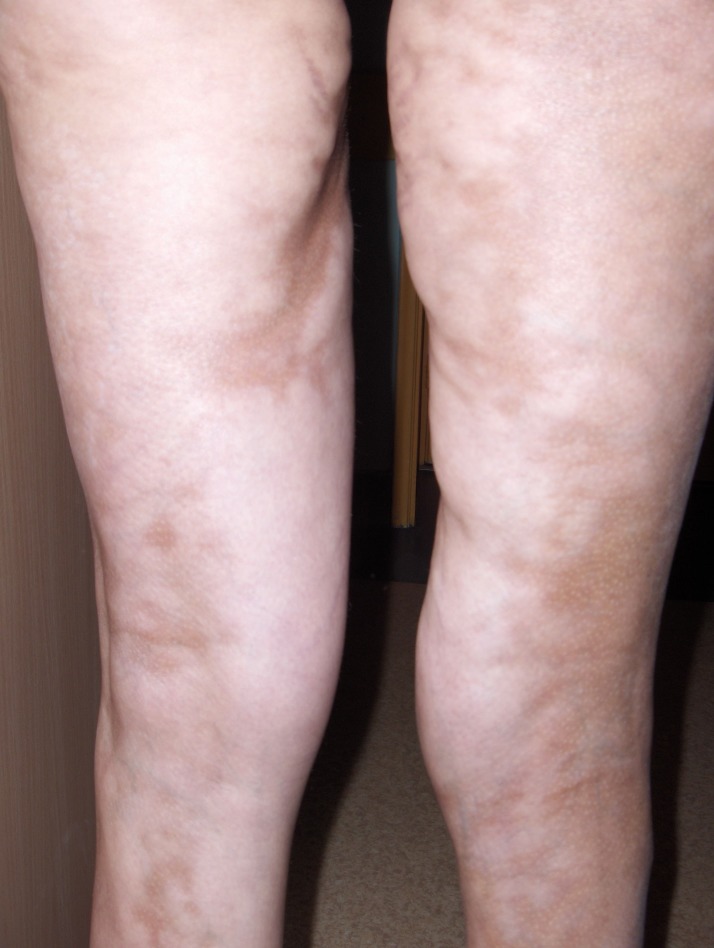
pansclerotic lesions with important atrophy of the underlying muscles and immobilization in semi-flexion of the fingers of both hands and first, with the absence of cutaneous fold; severe limitation of the mobility of the wrists

**Fig.6:**
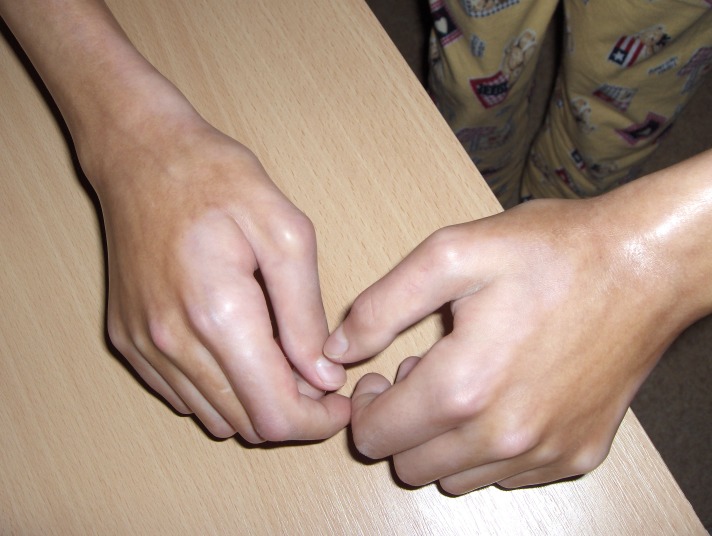
extended sclero-atrophic lesions with indurations and atrophy on the surface of the legs, with sclerotic bands extending over the knees to the proximal thighs

Seven months later she returned to our department, with severely aggravated disease. At this time the clinical examination showed an underweight patient (39kg for 160cm height), with extended sclero-atrophic plaques on flanks and chest, with indurations and atrophy of the left breast and sclerosis involving all the surface of forearms and elbows up to distal third of the arms, as well as the surface of the ankles and legs, with sclerotic bands extending over the knees to the proximal thighs. Fibrous bands extended to the left shoulder, with impairment of arm movements. (**Figures 4**-**6**). The fibrosis had progressed deep, involving soft tissue and fascia, with important atrophy of the underlying muscles and immobilization in semi-flexion of the fingers, fixation of elbows at 90 degrees flexion, severe limitation of the mobility of the wrists and ankles and moderate limitation of extension of the right knee. Gait was difficult; the patient could not write, hold objects in hands or care for herself unassisted. Notably, the face was spared. 

The laboratory tests showed persistent hypochromatic anemia (Hg=11.1g/dl), thrombocytopenia (Plt=96,000/μL), eosinophilia (Eo=750/μL; 14.9%), elevated ESR of 86 mm/h, hypoalbuminaemia with hypergammaglobulinaemia, positive rheumatoid factor, and highly increased anti-streptolysin O antibody (ASLO) titer. ANA and anti-Scl70 antibodies were still negative. No clinical nor paraclinical signs of further organ involvement were found. Antibiotic treatment was started with amoxicillin 2g/day for 10 days. After interdisciplinary consultation with rheumatology specialists, in view of the rapid progression of the disease, with high inflammatory activity and severe motor handicap, we opted for a therapy scheme combining monthly pulse-therapy with methylprednisolone 250mg/day i.v., over 4 days, continued with methyl-prednisolone p.o. 16mg every other day, methotrexate 7.5mg/week, piascledine, pentoxifyllin 300mg/day, and aspirin 75mg/day, associated with supportive potassium, magnesium, iron and folic acid supplementation. In the same time, the patient started a complex and intensive physio- and kinetotherapeutic rehabilitation program. 

This scheme was continued for 3 months. UVA phototherapy at 20J/cm² dose per session, 5 times/week was added during the last month.

Under this treatment, the evolution was favorable, with resolution of the inflammatory process, lack of appearance of new lesions, slight overall softening of fibrotic skin and improved motility of involved joints (gain of 20 degrees of extension in elbows and right knee, improvement of gait). In the same time laboratory parameters, including blood count, ESR, and ASLO titer returned to normal. Hypoproteinemia with hypoalbuminaemia (total proteins=5.8g/dL) was the only persistent disturbance, for which a hyper-proteic diet was recommended.

## Discussion

We present a challenging case of disabling pansclerotic morphea of childhood.

The diagnosis of this disease form may be difficult. DPMC may resemble at onset the plaque-type morphea or linear scleroderma and hence the diagnosis of the complete form, with its ominous prognostic implications might be established only late, once that pansclerosis with joint ankylosis and muscle atrophy occur [**5**]. Classification of DPMC is also still controversial. This form is considered by some authors as being the most severe variant of linear scleroderma; other authors, due to the involvement of all structures overlying the bone (sometimes even bone), place it in the group of deep morphea [**11**, **2**, **12**], while its tendency to expansion and generalized cutaneous involvement may ground its classification as a variant of generalized morphea.

Anemia, inflammatory biological syndrome, thrombocytopenia and eosinophilia present in our case are usually rare in plaque-type morphea, but are described relatively frequently in pansclerotic morphea [**13**, **14**, **15**]. These parameters correlate well with the activity of the disease, and may be used to monitor the evolution. A similar role has been recently reported for the serum levels of IL-2 soluble receptors [**16**].

During the diagnostic work-up of DPMC, it is important to differentiate this aggressive form of localized scleroderma from progressive systemic sclerosis. Absences of Raynaud phenomenon and of proper sclerodactylia, with characteristic sparing of the tip of the fingers are useful clues for the diagnosis of this disorder in the group of circumscribed scleroderma. 

Absence of organ involvement must be regularly checked, as late progression to visceral fibrosis in the course of DPMC has been reported [**17**].

The etiology of DPMC remains unknown, as well as the factors influencing its possible galloping evolution. In our case severe aggravation of the disease occurred explosively, over few months, on patient’s self-decided discontinuation of all treatment. The role of the reported airways infection, associated with high levels of ASLO titer in the sudden progression of the disease may be speculated, based on the well described correlation between streptococcal infections and rheumatic, autoimmune diseases [**18**].

The risk on long term of DPMC is poor and the complications to fear are: the possible generalization of cutaneous sclerosis, with irreversible ankylosis; progression of lesions to the face, with secondary mutilation due to scarring alopecia, ectropyon, spontaneous amputation of ears, dental malpositions; osteolysis with distal amputations of phalanges; cachexia; transformation into systemic sclerosis; the development of cutaneous trophic ulcers with superinfection and possible septicemia; neuro-compressive syndromes; development of squamous cell carcinoma.

Besides these, the complex side-effects of treatment, usually including active immunosupressors, must be taken into account [**19**].

The lethal risk is related to chronic restrictive pulmonary disease in the event of extension of sclero-atrophic lesions of the thoracic wall; to superinfection of cutaneous ulcers with secondary septicemia; to secondary cancers developing on the areas with cutaneous sclerosis and ulcers and to progressive cachexia [**19**].

In our patient, important motor handicap developed over 7 months, so that the 19-year-old girl was incapable of self-care and of self-support. Chronic ulcers, neural entrapment syndromes and cutaneous carcinogenesis may occur later in her case, and attentive monitoring should be performed.

Pansclerotic morphea, with all possible complications represent a challenge for the management. To date, there is no standard strategy of treatment, as no therapy has been consistently proven to be efficient in stopping the progression of the disease [**19**, **8**, **9**, **10**]. Combined treatment with methyl-prednisolone in pulse-therapy, methotrexate and phototherapy with UVA are described as the most efficient methods. Corticosteroid pulse- therapy is important for the strong anti-inflammatory and immunosuppressant effect, in highly active phases of the disease, with less of the risks of adverse reactions associated to long term corticoid therapy. Methotrexate is assumed to inhibit the synthesis of proinflammatory cytokines (IL-2, 4, 6, 8) and the fibrosis process; however, used alone it has only a reduced effect on the course of the disease [**20**, **21**, **22**, **23**]. The use of low doses of methotrexate associated with pulse-therapy with methyl-prednisolone has the most favorable risk/benefit rapport [**24**]. Phototherapy with UVA has anti-inflammatory, immunosuppressive but also antifibrotic effects by collagenases induction, defensins synthesis and induction of lymphocytes apoptosis [**25**].

For our patient, it was important to take an interdisciplinary approach, with the cooperation of dermatologists, rheumatologists, gastroenterologists and kinetotherapy specialists. The combined therapy of steroid pulse-therapy, low-dose of methotrexate and with low dose UVA phototherapy (20j/cm²) supported by an intensive program of physio- and kinetotherapy had an encouraging result, with prompt reduction of inflammatory activity, stop of the disease progression and even slight reversion of cutaneous fibrosis. For the further management on longer term of this patient, however, the well known side effects of corticotherapy and methotrexate as well as the susceptibility to cutaneous carcinogenesis must be kept in mind.

Other therapy options of morphea are reported in literature, with different rate of success as immunomodulators and antifibrotic agents. These include antimalarials, colchicine, cyclosporine, tacrolimus, penicillamine, intravenous immunoglobulins, interferon gamma, and recombinant human relaxin [**26**].

Newer drugs also wait for confirmation in larger clinical studies. Thus, a recent drug tested in pansclerotic forms of morphea is sildenafil, a specific phosphodiesterase type 5 inhibitor which determines an increase of nitric oxide. Although it isn’t approved as a first therapeutic line, it is assumed that sildenafil could at least partially prevent the evolution of the disease. It was proved to be efficient for ulcers and for Raynaud syndrome. Also, it was proved to have antitumoral effect *in vitro *[**20**, **27**]. Bosentan – an endothelin receptor antagonist - has been recently reported to have beneficial effects in ulcerative forms of DPMC [**28**]. Targeting the transforming growth factor-beta (TGF-beta), a key mediator of fibroblasts activation and tissue fibrosis, may also be a promising strategy, as shown by in vitro studies [**29**].

## Conclusions

We presented a severe case of disabling pansclerotic morphea of childhood, with several particularities, such as: unusual polymorphous clinical picture, mixing plaque, linear and pansclerotic, circumferential lesions; symmetric involvement of all limbs with centripetal progression; explosive evolution leading to invalidity, after the self-decided discontinuation of all treatments and possibly related to a streptococcal infection. 

This article underlines the importance of a severe disorder, with cutaneous onset but with implications at systemic level, way beyond the “skin-depth”, and with a potential lethal prognosis. The early diagnosis is essential for initiation of proper therapy and interdisciplinary approach is indispensable for the proper management of these difficult cases. In the lack of established efficient therapy and facing a disease with unpredictable evolution, a major place belongs to attentive care, physio-kinetic rehabilitation and careful monitoring of these patients for prevention of complications, especially those that bear a vital risk like skin cancers, ulcers superinfections, and restrictive pulmonary disease.
